# Dopaminergic therapy in aphasia

**DOI:** 10.1080/02687038.2013.802286

**Published:** 2013-06-14

**Authors:** Sumanjit K. Gill, Alexander P. Leff

**Affiliations:** 1 Department of Medicine, Watford General Hospital, Watford, UK; 2 Institute of Cognitive Neuroscience, University College London, London, UK; 3 Department of Brain Repair and Rehabilitation, Institute of Neurology, University College London, London, UK

**Keywords:** Dopamine, Aphasia, Review, L-dopa, Bromocriptine

## Abstract

**Background:**

The dopaminergic system is involved in a wide range of cognitive functions including motor control, reward, memory, attention, problem-solving and learning. This has stimulated interest in investigating the potential of dopaminergic drugs as cognitive enhancers in aphasic patients.

**Aim:**

To discuss the evidence for the use of dopaminergic agents in patients with aphasia. Levodopa (L-dopa) and the dopamine agonist bromocriptine are the two drugs that have been trialled to date. We discuss, in some detail, the 15 studies that have been published on this topic from the first case report in 1988 to the present (2012), and assess the evidence from each.

**Main contribution:**

In addition to summarising the effectiveness of the drugs that have been tried, we examine the possible cognitive mechanisms by which dopaminergic drugs may act on language function and aphasia recovery. Given the wide range of dopaminergic drugs, it is surprising that such a narrow range has been trialled in aphasic patients. Important lessons are to be learned from published studies and we discuss optimal trial designs to help guide future work.

**Conclusions:**

The evidence for the efficacy of dopaminergic agents in aphasia therapy is mixed. Further trials with better tolerated agents are required. Optimal trial designs with appropriate control groups or blocks should be used. The mechanism of action is unclear, but at the cognitive level the evidence points towards either (re)learning of word-forms or their improved retrieval.

Levodopa (L-dopa) and the dopamine agonist bromocriptine are the two main drugs that have been trialled to date in patients with acquired aphasia. These studies were prompted by the observation that some Parkinsonian patients noted improved speech function following L-dopa therapy (Quaglieri & Celesia, 1977). Subsequent studies in patients with Parkinson's disease have shown that dopamine therapy can modulate the motoric aspects of speech: articulation and phonation, see (Goberman & Coelho, 2002) for a review of this literature; as well as the more linguistic aspects, verbal fluency (Gotham, Brown, & Marsden, 1988) and even sentence comprehension (Grossman et al., 2001). In post-stroke aphasia, the first reported use of dopaminergic therapy was bromocriptine used in a 62-year-old patient with a severe transcortical motor aphasia following a left frontal intracerebral haemorrhage 3.5 years prior to therapy. The bromocriptine caused a small improvement in his confrontation naming (5–10%) with a more marked improvement of the fluency of his spontaneous speech, and pauses between utterances were reduced by 24% (Albert, Bachman, Morgan, & Helm-Estabrooks, 1988). Single-case studies are often difficult to interpret, but if more than one period of “off–drug” is included, this helps. In this case, the patient's language function returned to baseline after cessation of therapy.

Before examining the rest of the literature, we will provide a quick pharmacological and anatomical resume of the dopaminergic system in humans. Dopamine is a monoamine neurotransmitter whose function was first characterised in 1958 (Benes, 2001). It is synthesised from L-3,4-dihydroxyphenylalanine (L-dopa), 95% of which is turned into dopamine by the action of the enzyme dopa-decarboxylase. The remaining 5% is then converted to noradrenaline and acts on adrenergic receptors. Dopamine acts on five subtypes of the dopamine receptor; however, these receptors fall into two main groups based on morphological grounds: D2-like (D2–D4: primarily located in the striatal neurons) and D1-like (D1 and D5: primarily located in the cortical neurons) (Civelli, Bunzow, & Grandy, 1993). Endogenous dopamine is mainly produced by two paired, midbrain nuclei which are situated close to each other: the ventral tegmental area (VTA) and substantia nigra (SN). Both project to different parts of the striatum and indirectly affect the cortical neurons via cortico-basal ganglia circuits, although the VTA also projects directly to prefrontal cortex (Figure 1(a)). A widespread subpopulation of cortical neurons also have dopamine receptors, so exogenous dopamine, particularly the receptor agonists and antagonists, have two main routes to affect the behaviour; either directly, or through existing cortico-basal ganglia circuits. Dopamine has been implicated in modulating a whole range of cognitive functions: motor control, reward, memory, attention, problem-solving and learning (Alexander, DeLong, & Strick, 1986) and could potentially affect language recovery through any or all of these.

The relative affinity of each dopamine agonist for each receptor subtype provides both its therapeutic effect and unwanted side effects. For example, the motor improvement seen in Parkinson's patients is attributed to the stimulation of D_2_ receptors in the caudate and putamen, as are the unwanted end of dose deterioration and dyskinesias (Kvernmo, Hartter, & Burger, 2006). Perhaps surprisingly, the only dopamine agonist trialled in aphasic patients to date is bromocriptine. Bromocriptine is primarily a D2 receptor agonist with some D1 receptor antagonist properties (Kvernmo et al., 2006).

## TRIAL EVIDENCE

Interest in dopaminergic effects on language recovery stemmed from the single-case study by Albert et al. (1988) and discussed above. The better trials have tended to be those with larger numbers and included a placebo-controlled arm. Without a placebo arm, patients always know when they are on the active drug so placebo effects can muddy the picture. Even with a placebo, not all trials blind the patient to this information (open-label trials). Some form of control arm or block is always preferable because most patients are on an upward recovery curve. Even though “spontaneous” recovery slows three months post infarct (Lazar et al., 2010; Lomas & Kertesz, 1978), therapy-driven improvements in aphasia are reported in the chronic phase, even decades after the stroke occurred (Moss & Nicholas, 2006; Price, Seghier, & Leff, 2010). All but three of the trials reported here were performed in the chronic phase (defined as >6 months post-stroke); there is good evidence that treatments aimed at improving language outcomes are effective in this latter time period (Allen, Mehta, McClure, & Teasell, 2012). The 15 studies in aphasic patients (the vast majority with stroke) covered by this review are summarised in Table 1. Relevant studies were found by searching PubMED and Google Scholar using the following terms: aphasia, anomia, bromocriptine, dopaminergic, drug therapy, L-dopa, levodopa and rehabilitation. Two single cases, both reported in books, were highlighted by one of the referees. We will discuss them in chronological order.

**TABLE 1:** Summary of the 15 studies covered by this review

*Title, 1st author, year*	*Trial type*	*Drug and dose*	*No. Pts*	*Aphasia type and chronicity*	*Main outcomes, comments*
Pharmacotherapy for aphasia. Albert (1988).	Single case, no placebo, ABA design.	Bromocriptine, 15 then 30 mg	1	Transcortical motor aphasia. 3.5 years post-stroke.	Paired with therapy. Reduced hesitancy, decreased paraphrasias and increased naming ability. Return to baseline after cessation of therapy.
The effects of bromocriptine on speech and language function in a man with transcortical motor aphasia. MacLennan, 1990.	Single case, placebo-controlled, single blinded, AB design.	Bromocriptine 2.5 mg increased to 15 mg	1	Transcortical motor aphasia. 4 years post-stroke.	An increase in no. words and correct information units, but no statistical analysis made. Changes could be due to practice or carry-over effects.
Bromocriptine treatment of non-fluent aphasia. Gupta (1992).	Two cases, no placebo, AB design.	Bromocriptine, escalated dose: 10 mg, 30 mg	2	1 = Broca's aphasia; 1 = transcortical motor aphasia. 18 months and 10 years post-stroke.	Drug alone, not paired with therapy. Fluency of speech improved in both. In the second case worse on 30 mg and improved when dose deescalated to 10 mg.
An open label trial of bromocriptine in non-fluent aphasia. Sabe (1992).	No placebo, ABA ramp-up, ramp-down design.	Bromocriptine, up to 60 mg/day	7	4 = transcortical motor aphasia; 2 = Broca's aphasia; 1 = global aphasia. > 1 year post-stroke.	Paired with therapy. An improvement seen in those who were moderately affected (4:3) with increased word finding and verbal fluency increasing on drug compared with baseline and then decreasing as the drug was withdrawn.
A randomised, double blind, placebo controlled study of bromocriptine in non-fluent aphasia. Sabe (1995).	Randomised*, double-blind, placebo-controlled, crossover design.	Bromocriptine, up to 60 mg/day	7	2 = Broca's aphasia; 3 = transcortical motor aphasia; 2 = anomic aphasia. > 1 year post-stroke.	No benefit measured over placebo in hesitancy, verbal naming, verbal fluency, content words or content units. High rate of side effects in therapy block (57%) compared with control block (0%).
Bromocriptine treatment of non-fluent aphasia. Gupta (1995).	Double-blind, placebo-controlled, crossover design.	Bromocriptine, up to 15 mg/day	20	7 = transcortical motor aphasia; 4 = Broca's aphasia; 9 = “mixed anterior aphasia”. > 1 year post-stroke.	Drug alone, not paired with therapy (therapy was not allowed during the trial). No improvement in speech fluency, language content, overall aphasia severity or non-verbal cognitive problems.
Bromocriptine is ineffective in the treatment of chronic non-fluent aphasia. Ozeren (1995).	No placebo, AB design.	Bromocriptine, 10–25 mg/day	4	2 = Broca's aphasia; 1 = transcortical motor aphasia; 1 = global. 24 to 35 months post-stroke.	No significant improvements on “aphasia tests” (a speech sample rated on an ordinal scale of three points). Outcome measure almost certainly not sensitive to change.
Transcortical motor aphasia. Berthier (1999).	Single case, no placebo, AB design.	Bromocriptine, titrated to 20 mg/day	1	Transcortical motor aphasia. Bilateral striatocapsular strokes (12 and 5 months prior to study) with Parkinsonian features.	Patient improved markedly on spoken picture description and aphasia quotient of the WAB. Mixture of improvements in Parkinsonian (motoric) and word finding aspects of speech.
An open label trial of bromocriptine in non-fluent aphasia: a qualitative analysis of word storage and retrieval. Gold (2000).	No placebo, ABBA design.	Bromocriptine, 15 mg/day	4	2 = Broca's aphasia; 2 = transcortical motor aphasia. 7 to 78 months post-stroke.	Drug alone, not paired with SALT. A significant improvement in word retrieval for all four on bromocriptine therapy with three experiencing decreases after withdrawal.
Bromocriptine and speech therapy in nonfluent chronic aphasia after stroke. Bragoni (2000).	Double-blind placebo-controlled, but not randomised (placebo 1st block; bromocriptine 2nd block).	Bromocriptine, 30 mg/day	11(5#)	9 = Broca's aphasia; 2 = global aphasia. 6 to 96 months post-stroke.	Improved dictation, reading-comprehension, repetition and verbal latency but only 5/11 completed the study. Supposedly double-blind but high level of side effects. Placebo-controlled but not block-randomised.
Effects of bromocriptine in a patient with crossed non-fluent aphasia: a case report. Raymer (2001).	Single case, no placebo, ABABA design.	Bromocriptine, escalating dose up to 20 mg/day	1	Transcortical motor aphasia. NB: right frontal stroke involving IFG. 2 months post-stroke.	Improved verbal fluency sustained during withdrawal phases—this could have been due to spontaneous recovery.
A clinical trial of bromocriptine for treatment of primary progressive aphasia. Reed (2004).	Randomised, double-blinded, placebo-controlled, crossover.	Bromocriptine, 22.5 mg per day	6	Primary progressive aphasia (mean age 66.8 years).	Increased mean length of utterance but no effect on fluency or naming. Suggests a slowing of decline of the motoric aspects of speech.
A randomised double blind trial of bromocriptine efficacy in non-fluent aphasia after stroke. Ashtary (2006).	Randomised, double-blind, placebo-controlled.	Bromocriptine, up to 10 mg/day	38	Non-fluent aphasic patients, Persian speakers, in the “acute” phase.	Patients in both groups (placebo and bromocriptine) improved on all measures. No significant differences between the groups
New approach to the rehabilitation of post-stroke focal cognitive syndrome: effect of levodopa combined with speech and language therapy on functional recovery from aphasia. Seniow (2009).	Randomised, double-blind, placebo-controlled.	L-dopa, 100 mg/day. Used in a phasic manner and paired with SALT	39	Any aphasia subtype. Acute phase (∼5 weeks post-stroke). In-patients	Increased naming and repetition on L-dopa compared with placebo. First study to attempt a sub-group analysis on lesion site (patients with “anterior” lesions responded better to L-dopa).
Crossover trial of subacute computerised aphasia therapy for anomia with the addition of either levodopa or placebo. Leeman, Laganaro, Chetelat-Mabillard, and Schnider (2011).	Randomised, double-blind, placebo-controlled, crossover.	L-dopa, 100 mg/day. Used in a phasic manner and paired with computerised therapy and SALT.	12	2 = Broca's; 2 = Wernicke's; 6 = anomic; 1= conduction. Acute (∼7.5 weeks) Post-stroke (9) or traumatic brain injury (3). In-patients.	Significant improvements on the treated items from the computerised therapy battery but no interaction with drug/placebo block.

* Although reported as randomised (block randomised), all 7 patients were randomised to drug first then placebo. See text for further comment.

# High drop-out rate (54%). Only 5 completed the study.

**Figure 1.**
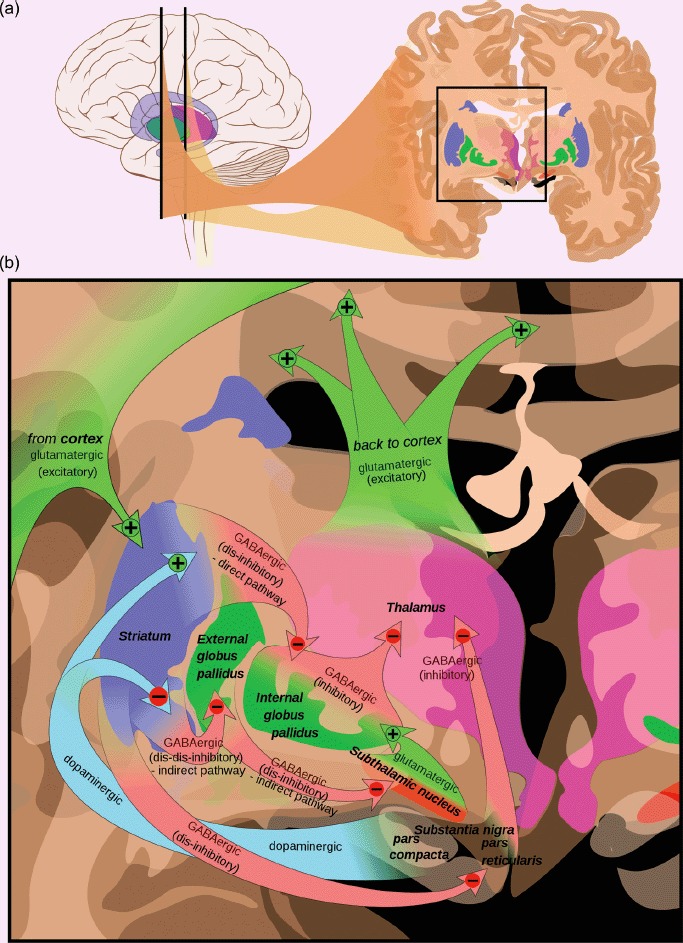
(a) The two main dopaminergic nuclei are close to each other in the midbrain with the substantia nigra projecting to the striatum and the ventral tegmental area projecting to both the nucleus accumbens (part of the ventral striatum) and the frontal cortex. (b) The nigrostriatal pathway affects cortical function indirectly through a series of cortico-basal ganglia circuits. The substantia nigra (pars compacta) projects to the striatum (caudate and putamen in blue). The main output of the striatum is to the globus pallidus (green) and thence to the thalamus (pink) and cortex (green arrows). The cortex feeds back to the striatum (green arrows) to close the loop on the cortico-basal ganglia circuits. There are also dopaminergic receptors on many cortical neurons (not shown). http://en.wikipedia.org/wiki/File:Basal_ganglia_circuits.svg. Mikael Häggström, based on images by Andrew Gillies.

Bromocriptine has been the drug of choice in 13 of the 15 studies. In Parkinson's disease, a dose range of 10–30 mg is usual, although bromocriptine has somewhat fallen out of favour with movement disorder specialists who prefer the newer dopamine agonists such as pramipexole and ropinirole. While bromocriptine is considered to be an effective treatment for Parkinson's disease, movement disorder specialists generally avoid all ergot-derived dopamine agonists because of the risks of lung fibrosis and heart valvulopathies (Dr Prasad Korlipara, Personal communication, 8 March 2013; Andersohn & Garbe, 2009). In order to minimise the occurrence of side effects, most studies employed an escalating dose regime up to 10 mg/day. Most titrated to between 10–30 mg but one group preferred to escalate to 60 mg. In the latter of their two studies, four of the seven patients taking part (57%) suffered side effects of nausea, dystonic movements or a lack of energy in the bromocriptine block (Sabe, Salvarezza, Garcia Cuerva, Leiguarda, & Starkstein, 1995). Another study with 11 patients titrated to 30 mg/day had a 63% incidence of side effects (Bragoni et al., 2000).

In 1991, MacLennan reported a case similar to the Albert one, although they used an AB and not an ABA design, which makes it difficult to interpret. Like the Albert case, there was no statistical treatment of the behavioural data, but there were two methodological improvements: a placebo was used with the subject blinded to the order of placebo/bromocriptine therapy, and a simple motor reaction time test was used to gauge any motoric effects of bromocriptine. There was no convincing effect of bromocriptine on motoric reaction time, naming or word fluency. The patient produced about 50% more words on a test of spoken picture description, with this effect continuing into the drug withdrawal phase. It is difficult to interpret this as the drug effect could be confounded by practice effects. The authors themselves concluded that the study was negative although, interestingly, the patient did not agree (MacLennan, Morley, & Brookshire, 1991).

In 1992, Gupta and Mlcoch (1992) went one better than the papers by Albert and MacLennan and reported a positive effect of bromocriptine in two aphasic patients with nonfluent aphasia in the chronic phase. Using an AB design (but no placebo block), both were started on bromocriptine and escalated from 10 mg to 30 mg/day. The main outcome measure was mean length of utterance, derived from an analysis of spoken picture description. The first patient showed some improvement in fluency at 10 mg and marked improvement at 30 mg, but it is difficult to rule out a simple time-effect (they perhaps should have tested him with an ABA design like Albert). The second patient's results were more interesting; he improved at 10 mg, got worse at 30 mg and improved again at 10 mg suggesting that, if the effect was due to the drug, the patient may have been affected by the inverted “U” shape curve described in drug-based cognitive enhancement (Husain & Mehta, 2011).

In the same year, Sabe, Leiguarda, and Starkstein (1992) reported an open-label trial in seven patients with mixed aphasia type and severity according to the Western Aphasia Battery (WAB) (Kertesz, 1982): four moderate, three severe. The trial was effectively an ABA design with language tests at eight consecutive time points (every 2 weeks) as the dose of bromocriptine was ramped up and then down (0, 15, 30, 45, 60, 30, 15, 0 mg). This sort of design is reasonable as any time effects or familiarity with the language tests should be dissociable from drug effects when the dose ramps down. In theory this type of study can be blinded (patients blinded to the dose), but it looks like this was not the case. Conventional Speech and Language Therapy (SALT) was administered as well. One has to interpret the results with caution as the numbers were small, so inferences to the reference population (all aphasic patients of this type) must necessarily be limited, but they did show convincing dose effects in the moderate group's lexical index scores (a measure of correct word production in a composite picture description task), verbal fluency and pauses >3 seconds (a measure of speech fluency). So some patients improved on a mixture of tests that could reflect improved word retrieval, spoken fluency (motor) or non-specific effects such as attention. The study was not placebo-controlled or blinded, so the authors followed it up with a better designed study that was published three years later (Sabe et al., 1995). This also had the same number of patients in the chronic phase but who were less variable in terms of severity than the original study, all being in the moderate range on the WAB. A similar escalation of bromocriptine as in the 1992 study (6 weeks on drug) was used, but this time with no ramp-down, just a 3-week washout and then moving into the next block (6 weeks on placebo). Language outcome measures were taken weekly. In theory, this was a better designed study than the 1992 one; however, inexplicably, all patients were allocated to the “bromocriptine first” group. The chances of this happening are 0.5^7^ or 1/128, so either they were very unlucky or something went wrong with their randomisation procedure. In small studies, it is a good idea to use block randomisation to avoid this (Lachin, Matts, & Wei, 1988). While they did employ a crossover design, the failure of random allocation of block order meant that time effects were conflated with treatment effects although the study was negative, with no significant drug (or time) effects found for any of the language outcome measures.

In the same issue of *Neurology*, Gupta et al. (1995) reported the findings of their larger study with 20 patients at least 1 year post-stroke, median = 3 years (Gupta, Mlcoch, Scolaro, & Moritz, 1995). This was a follow-on study from their 1992 paper with two patients. They escalated the bromocriptine dose by 5 mg a week to 15 mg which was given for 6 weeks followed by a 6-week washout. A crossover design was employed with patients randomised to placebo first (*n* = 9) or bromocriptine first (*n* = 11). SALT was prohibited during the trial so this was the first group study to investigate dopaminergic effects independent of concomitant SALT. The trial was well conducted; there were five evaluation periods, three at times when subjects were drug/placebo-free so test-learning effects could be evaluated (and discounted). Outcomes included language assessments (WAB, Boston naming tests and a transcription of conversational speech) and cognitive assessments (Wechsler Memory Scale, Raven's Progressive Matrices and the Rey–Osterrieth Figure). There were no significant effects (drug vs. placebo) on any of these outcome measures. The year 1995 was a busy one and saw the publication of a third study that was also negative, although only four patients took part (Ozeren, Sarica, Mavi, & Demirkiran, 1995). Bromocriptine was given initially at a dosage of 10 mg/day, and then 25 mg/day (no placebo). The main outcome measure was a stratified version of the Aphasia Test for Turkish Citizens where a speech sample was analysed and rated as either “A” (absence of speech), “B” (utterances with polymorphic syllabic fragments and phonemic jargon) or “C” (marked dysarthria and frequent stops within utterances). This outcome measure is almost certainly insensitive to the changes likely to be found with a drug therapy. Bromocriptine was found to be ineffective.

A further case report of similar design but with superior outcome measures was published as a book chapter (Berthier, 1999). This was an interesting case as the patient, who suffered bi-hemispheric, subcortical strokes 7 months apart, had Parkinsonian features as well as word-finding difficulties. After baseline assessment, there was a clear improvement in the amount of speech produced (53 words in 120 seconds of spoken picture description compared with six at baseline) with a more modest improvement (57 vs. 50) in confrontation naming. Some other test scores remained static suggesting some domain specificity, but with the lack of a placebo control, and thus any attempt at blinding, any effects beyond the clearly impressive motoric ones, are hard to interpret.

Gold, VanDam, and Silliman (2000) reported positive effects of bromocriptine in a small (*n* = 4, no placebo) trial of ABBA design (Gold, VanDam, & Silliman, 2000). Drug therapy was not paired with SALT. They specifically targeted anomia and used a bespoke anomia test with 420 items split into six lists of 70 matched items, (parallel forms), which were computer-delivered. Reaction times were measured and the authors also calculated a retrieval and storage quotient based on an analysis of error type. They found that all four subjects improved their retrieval quotients on the drug (with “large” effect sizes on Cohen's *d* (0.74–3.4)). These dropped back towards baseline once the drug was ramped down, in three out of the four subjects. If anything, the effect on storage was in the other direction (worse on drug) with no consistent effects on reaction times. There were no non-language tests.

Bragoni et al. (2000) used escalating doses of bromocriptine in 11 patients to reportedly good effect, but there were several major flaws. The study was placebo-controlled and tablet treatment was paired with SALT, but there was no block randomisation, so all the patients received placebo first and then bromocriptine (each block = 60 days). The authors claim the study was double-blinded, but it is not clear how the study design was concealed from the assessing therapists. Side effects were common with four patients dropping out because of these. Of the five that completed the study, three had nausea despite domperidone cover. Drug studies with a high incidence of side effects are likely to unblind subjects. Statistically significant improvements were seen in several language measures, but in all these analyses, the data following the drug and therapy block were compared with the baseline time point and not with the data collected after the placebo and therapy block, e.g. the two biggest effects were seen in reading aloud and dictation, but these improvements occurred after the placebo plus SALT block (26% and 43%, respectively) with only marginal improvement seen after the bromocriptine plus SALT block (29% and 47%, respectively). In short, despite the claims of the authors, the results do not so much advance the case of bromocriptine therapy as make a case for the effectiveness of SALT. Too great a dose of bromocriptine was used as side effects were prohibitively high.

In 2001, Raymer et al. (2001) used bromocriptine on a single-subject with transcortical motor aphasia due to a right frontal stroke. It was a no-placebo trial with an ABABA design. There was no mention of concomitant SALT. The authors included experimental (language) tasks that they predicted drug therapy would improve, and a control task (gesture), where they predicted no improvement. Multiple tests were carried out to establish a baseline and the main outcome was the slope (rate of improvement) in each of the four phases (the two drug blocks and the two following washout periods). The patient's ability to gesture did not improve significantly across any block. The biggest effect was on verbal fluency with the authors arguing that this was due to a bromocriptine as the rate of change on this measure was significant for the first drug block but not the baseline block or either of the washout blocks. However, the general trend in these control blocks was upwards and the appropriate comparison would have been to directly compare the slopes of the drug blocks with the control blocks, rather than comparing the slope of each block to the null (a flat line). In short, the study produced no convincing evidence of a drug effect.

All of the studies discussed so far had been carried out with patients suffering from post-stroke aphasia, but the next study was carried out on a group of six patients with primary progressive aphasia (Reed, Johnson, Thompson, Weintraub, & Mesulam, 2004). It is a short report of less than 400 words, so details are slim, but it appears to be a well-designed, placebo-controlled, double-blinded, crossover trial with three main outcome measures: naming, word fluency and narrative language. The blocks were ∼12 weeks long and while all the patients declined over this period, there was a significant reduction in this decline for the bromocriptine block for the mean length of utterance score (part of the narrative language score), suggesting a beneficial effect of bromocriptine on the motoric aspects of speech.

The largest bromocriptine trial is the most recent one which included 38 patients (Ashtary, Janghorbani, Chitsaz, Reisi, & Bahrami, 2006). This study recruited subjects from a single site in Iran and was carried out in the acute phase. Subjects were randomised by coin toss into the placebo or drug group. Coin-tossing is not a recommended method for small trials as this can sometimes result in an allocation sequence leading to groups that differ, by chance, quite substantially in size or in the occurrence of prognostic factors (Altman & Bland, 1999); although that did not appear to be the case with this study. A low dose of bromocriptine (10 mg per day) was used and no side effects were reported. No mention was made whether concurrent SALT was received by the patients. Performance on seven language tests and a control task (gesture) was measured before and after therapy. The patients in both groups improved on all eight outcome measures with no significant differences between the two groups; that is, there were significant time effects but no significant drug vs. placebo effects.

The two most recent studies used L-dopa rather than bromocriptine. The first was a well-designed, two-group study in 39 patients in the acute phase, on average 5 weeks post-stroke. Tablet therapy was paired with SALT (Seniow, Litwin, Litwin, Lesniak, & Czlonkowska, 2009). L-dopa was used in a phasic manner, that is, 100 mg (Madopar 125) was given 30 min prior to a SALT session which lasted for 45 min and occurred five times a week for 3 weeks. Performance on 10 subtests of the Boston Diagnostic Aphasia Examination was measured before and after therapy. As with the Ashtary study, subjects in both groups improved significantly on all tests compared to baseline; however, there was a significant group (L-dopa > placebo) effect on confrontation naming and repetition. This seemed to be driven mainly by the patients with frontal lesions (*n* = 12). Unlike previous studies, no attempt was made to limit recruitment based on aphasia subtype.

The most recent study by Leeman et al. (2011) was also conducted in the acute phase ∼7.5 weeks post-stroke and also used L-dopa in a phasic manner to augment SALT. The SALT therapy was more complex as it comprised of standard therapy in the afternoon, ∼1 hour a day, with additional computer-assisted therapy in the mornings. This was a programme that presented pictures to patients which they had to name, but rather than name aloud, they had to type in the name of the item. The authors examined item-specificity of the trained items across each block. The study was a crossover one so each subject passed through drug and placebo blocks (block duration only 2 weeks). There was an impressive effect of the computerised therapy with naming accuracy improving on the trained list by 25% on average, but there was no interaction with drug/placebo block. Although the therapy was supposed to be timed with drug administration, it looks like some computerised sessions occurred in the afternoon. Given that the half-life of the active drug (L-dopa with benserazide) was 90 min, this may have led to under-dosing. The group was also small and mixed (three had head injury rather than stroke) although the crossover design should have helped reduce the effects of this somewhat. The authors’ conclusions to their study pretty much mirror our general view that, “The type of substance, dose, time since brain injury and the type and intensity of therapy and timing between drug administration and therapy are important factors that need more systematic exploration (2011, p. 47)”.

## DISCUSSION

Of the 15 studies discussed here, only seven reported a positive effect of dopaminergic therapy. Two of these were randomised controlled trials, but the overall impression is that the balance of evidence suggests no beneficial effect, on average, across the studies. A rather out-of-date Cochrane review dismissed all the evidence from any of the dopaminergic therapy studies, although it was published prior to the more modern and more rigorously controlled studies (Greener, Enderby, & Whurr, 2001). In terms of the inferences one can make with frequentist statistics, positive studies carry more weight than negative studies. As Fisher said, after a negative study, one can only “withhold judgment” (Dienes, 2011); but negative studies are harder to publish and one can't help but wonder if publication bias is at work here, especially for the smaller, uncontrolled studies (Dwan et al., 2008).

If we put these concerns to one side for now and concentrate only on the positive studies, can we discern any pattern of improvement that may give a clue as to how and at what level (in terms of cognitive models) dopaminergic therapy may be working? Given that all of the studies focused either mainly or entirely on speech output, we can perhaps use Levelt's simplified model of speech production to try and assay where the positive effects are manifest. Levelt divides speech output into four main serial processes: concept generation, word-finding, motor planning and articulation (Levelt, 1999). In terms of which aspects of speech or language function improved, the following were found: naming/word-finding (5 studies); verbal fluency (which taps into several components of Levelt's model, 2 studies); motoric aspects of speech (repetition, 3 studies). Of course, this type of analysis is dependent on how often the individual measures were used and there is no accepted, standardised way of choosing outcomes in aphasia research. Almost all of the studies measured naming ability. There have been few studies in normal subjects that are relevant, but in a landmark study, Knecht and colleagues investigated the effect of L-dopa in normal subjects on novel word-learning in a randomised, placebo-controlled study (Knecht et al., 2004). They used 100 mg of L-dopa in a phasic manner, timed with computer-based learning of new words (pseudowords) for pictures of familiar items. The L-dopa group had significantly enhanced speed, overall success and long-term retention of novel word-learning than the placebo group, and they did so in a dose-dependent manner (while all subjects in the L-dopa group received the same dose, in a clever twist, the authors performed a subanalysis using a median split to assess this group by weight; the lighter subjects did significantly better). They included appropriate tests to rule out non-specific effects of motor performance, arousal, attention or response bias. The evidence from this study suggests that task-dependent learning can be improved by dopaminergic therapy, although the mechanism could be via the mesolimbic reward system (Fiorillo, Tobler, & Schultz, 2003), prefrontal cortical circuitry underlying working memory (Williams & Goldmanrakic, 1995) or via enhancing classical long-term potentiation at the hippocampal–prefrontal synapses (Gurden, Takita, & Jay, 2000). Returning to the patient data, it is very difficult to say much about the language specificity of these effects as few studies had a “control” outcome (two used gesture) where one might expect no effect of therapy. No studies reported measures of more basic cognitive components such as attention or vigilance which are likely to impact on any task-specific measures, which is a shame as there is good evidence that the dopaminergic system modulates these (Husain & Mehta, 2011); however, taken together with the Knecht study, the positive evidence points towards either new encoding (relearning) of word form information, or its enhanced retrieval as the most likely mechanism of action of dopaminergic therapy.

Perhaps the biggest surprise is the lack of agents tried. Bromocriptine dominates the literature, yet it has a poor side effect profile both in terms of short-term administration (as shown in several of the trials reviewed here that tried to push the dose to 30 mg/day and beyond) and in terms of longer-term risks of cardiorespiratory fibrosis. This agent would be unlikely to find many takers in real-world clinical practice. L-dopa has more translational potential but its bioavailability means that it is best suited for phasic use, perhaps paired with ongoing SALT. Both the trials using L-dopa were in the acute phase, but there is no good reason to think it would not work in the post-acute phase. Lastly, we were surprised that there are no studies with the newer dopaminergic agents. A search of the US and EU clinical trial databases revealed only a single ongoing trial of Tolcapone (a COMT inhibitor) in aphasic patients with fronto-temporal dementia. Perhaps future studies will consider the non-ergot-derived agonists or reuptake inhibitors such as methylphenidate.

All drug studies are resource-consuming and take a lot of effort, so what lessons can be learnt from the studies reviewed here that might guide future work? Perhaps firstly we should say that, given the effects and effect sizes of behavioural therapy, future studies should pair dopaminergic drug therapy with behavioural therapy of some sort; a view that others have endorsed (de Boissezon, Peran, de Boysson, & Demonet, 2007). While we can probably draw a veil over bromocriptine, it would be nice to see studies with newer agents or with L-dopa in the chronic phase. In terms of study design, generally, double-blind, randomised, placebo-controlled trials are best. Well-designed single-case or case-series studies are useful, but care should be taken to control for placebo and time effects, so multiple baseline, ramp-up, ramp-down or multiple alternating blocks (ABA, ABBA) should be considered (Hilliard, 1993). For group studies, we prefer crossover designs as these deal effectively with inter-subject variance, but if a more traditional two-group (or more) design is to be used, then block randomisation and/or minimisation should be employed to reduce the risk of the groups becoming unbalanced on key variables such as time since stroke or severity (Altman & Bland, 2005). Given the high incidence of side effects in some studies, building in a de-escalation option in the study protocol will help keep some patients in the study side effect-free, albeit on a lower dose. This seems reasonable as higher doses of cognitive enhancing drugs do not necessarily lead to better performance (Husain & Mehta, 2011). Studying patients in the acute phase, when “natural” recovery curves are at their steepest is always a challenge, but can be done using more innovative techniques such as randomised N-of-1 designs (Edgington & Onghena, 2007). Husain and colleagues used this in a study of the dopamine agonist rotigotine in acute, post-stroke neglect to good effect (Gorgoraptis et al., 2012). Finally, what to measure? It is probably not a good idea for language outcomes to be fixed for all aphasia studies, as these will need to be tailored to the impairments and disability profile of the participants, which will necessarily vary from study to study. Standardised tests that are sensitive to change, such as the Comprehensive Aphasia Battery (Swinburn, Porter, & Howard, 2004), are best. Non-language tests should also be included: probably a test of motor function (as the main function of dopamine is on the motor system) and also a test of attention. Lastly, patient-reported outcome measures should be used if possible because if we are to hope for clinical translation to proceed from clinical trials, we will need some evidence that patients or their carers find our interventions useful as well.
